# Conservatism *Versus* Radicalism in Certain Dental Operations

**Published:** 1903-03

**Authors:** J. G. Palmer

**Affiliations:** New York


### CONSERVATISM *VERSUS* RADICALISM IN CERTAIN DENTAL OPERATIONS.[1]

BY DR. J. G. PALMER, NEW YORK.

MR. PRESIDENT AND GENTLEMEN,—When your committee invited me to appear before you, it was suggested that some subject which would interest the younger men would also be sure to attract the older ones.

I have chosen to speak on the conservative side as opposed to radical innovations, partly because I am not in sympathy with much of the latter, and partly because so frequently those who advocate more advanced theories take too much for granted, and do not go into those details which are so essential to success in any undertaking. One of the gentlemen, whose theories of "immediate root-filling" I am not in harmony with, has very truthfully said, "We have sometimes failed in accomplishing what another has claimed to be an easy matter, because that one has omitted from his description some detail which he took for granted we were conversant with." I want to give him credit for having been very careful in this respect. It is a fact, however, that very many are not careful to go into the minutiæ of their work, taking too much for granted. In this very thing, if those who are advocating new methods—or even describing what to them are old

[1] Read before the Academy of Stomatology, October 28, 1902.

methods—would have more care, I would regard them as being conservative in the sense that conservative means "guarding," or "protecting."

I shall speak briefly on what I consider the conservative side of root-filling, of extension for prevention, regarding the devitalization of sound teeth for crowning, and the use of gold in filling children's teeth.

In methods of devitalization of the pulp, and in treatment of abscessed teeth, or devitalized teeth which have never abscessed but have been devitalized for long periods, the difference between conservative and radical methods is most marked.

There are those who earnestly advocate what they term *immediate root-filling.* This, as I understand it, means that a devitalized tooth is opened, cleansed, sterilized, and filled at one sitting. On occasion, some who advocate this method have sought to qualify their statements, but in the main they "stand by their guns." Certainly this is a very radical method as compared with what we have been taught, both in our schools and in our experience.

The statement has been made by some that they do not hesitate to surgically devitalize,—remove the pulp and fill at once,—but rarely do they describe the "surgical method" in full.

Other than the use of cocaine or eucaine, and gradual approach to the pulp, and then pressure anæsthesia, the general method is the use of arsenic, and there there is a difference,—one introducing the medicine for twenty-four hours and then removing the pulp, and another allowing the medicine to remain a week or more until sloughing has occurred, and then removing the contents of the pulp-chamber. In any case, however, whether immediate devitalization of the pulp, arsenical application for twenty-four hours, or a lingering death, the radicals would fill at once upon removing the pulp. Perhaps in that I am somewhat "radical," for I sometimes *do* fill on such removal,—no matter how I have destroyed the pulp,—but on occasion I have had serious trouble by so doing. Probably it has been my own fault. I may not have removed the entire contents of the root-canals, etc., but the question under such circumstances comes home to me forcibly, Is this method right? Would it not be wiser to be more conservative, and after removing the pulp—especially when there is profuse hemorrhage—give the wounded parts a chance to heal; let Nature have control for a little while before sealing the canal? In the light of my own experience, I cannot always feel that it is right to *immediately* fill, even under apparently favorable circumstances.

If not then, how much more do I feel that it is best to be conservative in treatment under any other circumstances than those mentioned, unless where a simple plain fistula presents, with no destruction of the surrounding process. In such cases I do not hesitate to fill at once after injecting through the tooth and out of the fistula. In all other cases, however, it has always seemed to me, from the very nature of things, as well as from my own practical experience at the chair, that the more conservative practice would insure better results in the end and often save the patient much pain.

Dr. F. Milton Smith, of New York, says, in an able article read before The New York Institute of Stomatology, November 1, 1898, on this subject, "Our treatment being almost without exception the SAME in *all* cases of pulpless teeth." He undoubtedly has "the courage of his convictions," but to apply his method to cases where pus is discharging from the tooth, or to those cases where everything is dry but very offensive upon opening, seems so very radical, and has so often at my hands been productive of michief, that I cannot help feeling that a more conservative practice is the better one.

One of our prominent college professors, who is quite radical in his practice, said he always told his patients that there was a chance of trouble should he fill at once, and, having told them so, he would do as they desired. If they were willing to take the consequences he would fill immediately.

Others less conservative even than he, stating that they expect no trouble, and that none ought to occur except perhaps a little soreness for a day or two, do not give their patients this opportunity to decide, but go on and fill at once. Both classes, however, agree in this one thing: "if trouble ensues, lance the gums freely and all will be well.

My own experience in following this advice has been unfortunate. As stated before, perhaps I have not been as careful as I should have been, perhaps I did not cleanse the canals thoroughly. But *why* do they *sometimes* have trouble? Why does the gum need to be lanced if they have *absolutely* cleansed and sterilized? Is such a radical method, which makes but little provision, if any, for hours of torture should they occur, and which does occasionally cause such hours, to be preferred to a more conservative practice, which, at the hands of the large mass of operators, certainly reduces the chances of trouble, and has in reality less of torture? The only advantage of the former over the latter seems to me to be in the time saved. Is it wise to endeavor to save time at the very possible risk of pain and failure?

Note that I do *not* say that it is not possible to *frequently* fill immediately and have no trouble, but since the most ardent advocates of this method admit having trouble on occasion, and since we believe that with the more conservative method less trouble will ensue, it has always seemed to your essayist the safer and wiser plan to pursue. Remember, please, that Dr. Smith says his treatment is the *same* in all cases, "*almost without exception.*" He speaks of the method as the "positive" one.

I would not be understood as favoring a *prolonged* treatment, unless in very exceptional cases. I quite agree that as much harm may be done by excessive treatment as by any other practice. I would be understood, however, as favoring that method of conservative treatment which should gradually eliminate poisonous elements, believing that, if the same care in introducing medicines is exercised in this method that some practitioners use in the more radical work, the likelihood of any undue results would be reduced to a minimum.

The question of "extension for prevention" shows in a marked degree the difference between radical and conservative methods.

An advocate of the former method favors extension under any and all circumstances, claiming that it is the *only* certain and sure method of preventing future decay. Those less radical say they do not always extend, but generally defending their practice on the ground that they do not have the small approximal cavities to deal with in the West which some Eastern men claim to have.

It has always seemed to me that in this matter one's good judgment, common sense, and a proper understanding of each case and its surroundings should all be added to a conservative idea, to do for each case what that case demanded, and not make it an iron-clad rule that a cavity must always be extended hither and yon, until there is no possibility of contact, hence no possibility of decay. It by no means always follows, however. "Simply this and nothing more" does not insure against further decay, extension or not.

Referring especially to these small approximal cavities, an able editorial article in the *Items of Interest* for September says, "Dr. Black does not tell us why this recurrence is at the margins of the fillings. Why not elsewhere in that region which is prone to decay, and why not as a distinct new cavity independent of the filled area? Why, indeed, unless it be that the fault was along that margin and due to improper manipulation rather than to improper position." And a little farther on: "If the excursions of food suffice to render the axial angle immune to decay, Dr. Black should give us the scientific explanation of caries along the labial surfaces of the anterior teeth,—the area of all others most thoroughly cleansed with the tooth-brush." I do not feel that I could say anything more emphatic than Dr. Ottolengui has in the paragraphs just quoted.

I do certainly feel that the claims of the most ardent advocates of "extension for prevention" are so extremely radical that the young practitioner may well consider and investigate and experiment before accepting their theories as self-evident facts.

This matter was carefully considered at a meeting of the Second District Dental Society of New York, in Brooklyn, on the evening of March 11, 1901, as reported in the *Items of Interest* for May, 1901. After much discussion pro and con, the consensus of opinion was strongly against indiscriminate extension, especially below the gingival margins. While it is yet an open question, it is quite evident that the advocates of "extension for prevention" have used all their ammunition, for they dogmatically reaffirm their position, but adduce no new arguments in favor thereof.

In crown- and bridge-work the question of whether sound teeth, or teeth with only small cavities, should be devitalized or not, when intended to be used as piers, has been frequently discussed of late. The radical says that he does not hesitate under any and all circumstances to devitalize. He claims that the tooth will be much more satisfactory. He states that the pulp will die in the tooth any way after crowning, and that he is only anticipating and thereby saving trouble to all concerned. Perhaps he believes that "dead men tell no tales."

Having in mind cases which I have seen from the hands of workmen skilled in "gold and silver," who have followed this idea and devitalized sound teeth and crowned at once, I am firm in my conviction that the combination of circumstances was too much. I have seen a number of such cases where abscesses have formed, whether because immediately filled or not does not concern us now, but, had the teeth not been devitalized, had their life forces been conserved, the crowns so adapted that there would have been no irritation, my impression is that the results would have been better, that the teeth would have given no trouble, and the patients would have been saved the pain and annoyance of the abscesses plus the subsequent necessary removal of crowns or bridges to gain access to the pulp-chamber. That this subject admits of much discussion is true. I have seen cases where the action of the cement upon the tooth has been very irritating and resulted ultimately in the loss of the pulp. I have always believed, however, that this was due to some defect in preparing the cement, or in the cement itself. Those cements which harden thoroughly throughout do not seem to affect the teeth so readily, and where a lining of gutta-percha can be supervened it would seem that the tooth could be readily preserved.

As crown- and bridge-work is being more and more practised, this question seems to me to be one in which the conservation of the teeth should be most earnestly considered. My own antipathy to the ruthless destruction of the pulp in strong healthy teeth is so great that I cannot give with any degree of acquiescence the theory or practice of devitalizing such teeth merely to make piers, and I fail to be convinced that a sound tooth, even under crowning, must necessarily die.

"Should children's teeth be filled with gold?" was Dr. Ottolengui's theme before the New Jersey Dental Society at Asbury Park this summer, and, despite adverse criticism from many quarters, he championed that idea for all there was in him. It seemed quite a departure from our old-fogy, conservative ideas to say that gold was the best thing to use in cavities for children. All teaching and experience would seem to be opposed to this idea. Even the use of amalgam was opposed, and, if I remember aright, gutta-percha was inveighed against by the essayist.

My own experience and the experience and practice of many practitioners whom I know is strongly in favor of the use of both plastics and gutta-percha.

Consider the tender years, the extremely sensitive teeth, the time necessary, under most favorable circumstances, to insert a gold filling, and the care necessary to do so effectually, is it at all likely that such work in the hands of the average operator will succeed?

Often the child of even twelve or thirteen years comes to us with cavities so perilously near the pulp that any attempt to remove much, if any, of the floor of the cavity would cause trouble. Even pressure cannot be endured. Assuredly a soft material which can gently be introduced and which *will protect,* is to be preferred.

In a paper read before the Southern Dental Society of New Jersey, April 16, 1902 (*Items of Interest,* August, 1902), Dr. H. S. Sutphen says we should "first of all give 'mouth ease,' and secondly 'tooth preservation,' the second depending upon the first." How very apt this is, and how well it applies to our work for the little ones! They come to us so nervous and apprehensive of pain that to properly prepare and fill the cavities with gold would mean such a shock to the nervous system, such a dread of the dentist, that they would remember it for years. I doubt if they would ever forget it.

Is it wise to subject them to this exceedingly radical method when care and patience will help them tide over the period of such intense sensitiveness? Even the pressure of the pellet of cotton causes pain. Yet how often by care and attention we have been able to so fill such teeth with some of the various plastics at our command that in the course of time they have become stronger and healthier and able to receive the metal filling and do good service.

Do we not owe these young patients more attention, more care, than we could possibly give by subjecting them to the ordeal of a gold filling, be it ever so deftly and expeditiously done?

"Prove all things; hold fast that which is good," says Holy Writ. In all such discussions we earnestly desire to get at that which is good, and having done so, to hold on to it.

I have not offered any new arguments to-night, but if the way in which the old ones have been presented shall cause a discussion that will bring out some new ideas, or emphasize old ones, I shall feel that my effort has not been without avail.

